# Evaluation of Trends in Homeschooling Rates After Elimination of Nonmedical Exemptions to Childhood Immunizations in California, 2012-2020

**DOI:** 10.1001/jamanetworkopen.2021.46467

**Published:** 2022-02-02

**Authors:** Kavin M. Patel, SarahAnn M. McFadden, Salini Mohanty, Caroline M. Joyce, Paul L. Delamater, Nicola P. Klein, Daniel A. Salmon, Saad B. Omer, Alison M. Buttenheim

**Affiliations:** 1 Department of Internal Medicine, Yale School of Medicine, New Haven, Connecticut; 2 Yale Institute for Global Health, New Haven, Connecticut; 3 Department of Family and Community Health, School of Nursing, University of Pennsylvania, Philadelphia; 4 Department of Epidemiology, Biostatistics, and Occupational Health, McGill University, Montreal, Quebec, Canada; 5 Department of Geography and Carolina Population Center, University of North Carolina at Chapel Hill, Chapel Hill; 6 Kaiser Permanente Vaccine Study Center, Oakland, California; 7 Institute for Vaccine Safety, Departments of International Health and Health Behavior Society, Johns Hopkins Bloomberg School of Public Health, Baltimore, Maryland; 8 Department of Epidemiology of Microbial Diseases, Yale School of Public Health, New Haven, Connecticut; 9 Center for Health Incentives and Behavioral Economics, Perelman School of Medicine, University of Pennsylvania, Philadelphia; 10 Leonard Davis Institute of Health Economics, University of Pennsylvania, Philadelphia

## Abstract

**Introduction:**

In 2015, California passed Senate Bill No. 277 (SB 277) and became the first state in more than 30 years to eliminate nonmedical exemptions to mandatory childhood immunizations for school entry. One concern that emerged was that the law created an incentive for parents to remove children from brick-and-mortar schools to bypass the immunization requirements.

**Objective:**

To assess the trends in homeschooling rates after the elimination of nonmedical exemptions to the requirement of childhood immunizations for school entry.

**Design, Setting, and Participants:**

This preintervention-postintervention cross-sectional study calculated homeschooling rates as the number of students in kindergarten through grade 8 (K-8) enrolled through each of California’s 3 homeschooling mechanisms (independent study program, private school affidavit, and private school satellite program) divided by all K-8 students enrolled in the same academic year. Data on homeschooling rates were obtained from the California Department of Education. Interrupted time series analyses were conducted using a linear regression model in which the outcome variable was the percentage of students enrolled in a homeschool program before and after SB 277. Data were collected and analyzed from October 3, 2012, to October 2, 2019.

**Intervention:**

Passage of SB 277, which eliminated nonmedical exemptions to childhood immunizations for school entry.

**Main Outcomes and Measures:**

Homeschooling rates for K-8 students.

**Results:**

Among the students included in the analysis, the homeschooling enrollment for K-8 students in California increased from 35 122 students (0.8%) during the 2012-2013 school year to 86 574 students (1.9%) during the 2019-2020 school year; however, the implementation of SB 277 was not associated with an increase in the percentage of students enrolled in homeschooling programs in California beyond the secular trend. The increase in homeschooling was greatest for the lower grade levels: kindergarten homeschooling enrollment increased from 2068 students (0.4%) in the 2012-2013 school year to 10 553 students (1.9%) in the 2019-2020 school year, whereas the grade 8 homeschool enrollment rate increased from 5146 students (1.0%) in the 2012-2013 school year to 10 485 students (2.0%) in the 2019-2020 school year. Independent study programs accounted for 20 149 students (45.3%) of homeschooling enrollment, private school affidavits accounted for 19 333 students (43.5%), and private school satellite programs accounted for 4935 students (11.1%) during the 2015-2016 school year.

**Conclusions and Relevance:**

The findings of this study suggest that legislative action to limit nonmedical exemptions for compulsory vaccination for school entry is not associated with removal of students from classroom-based instruction in brick-and-mortar institutions.

## Key Points

**Question:**

What are the trends in homeschooling rates after the elimination of nonmedical exemptions to childhood immunizations for school entry in California?

**Findings:**

In this cross-sectional study of students in kindergarten through grade 8 in California, elimination of nonmedical exemptions to mandatory childhood immunizations for school entry was not associated with an increase in homeschooling rates.

**Meaning:**

The findings of this study suggest that elimination of nonmedical exemptions to the requirement of childhood immunizations for school entry is not associated with increases in the population of homeschooled children.

## Teaser

This cross-sectional study evaluates changes in homeschooling rates among kindergarten through grade 8 students before and after California’s Senate Bill No. 277 eliminated nonmedical exemptions to vaccination requirements in the state.

## Introduction

The so-called Disneyland measles outbreak of 2015 followed a decade-long decline in childhood immunization coverage rates in California.^1^ The state has required childhood immunizations as a condition of school entry since 1977.^2^ However, the mandate could be bypassed in 2 ways: (1) obtaining a nonmedical exemption based on a personal and/or religious belief or (2) filing for a medical exemption with the endorsement of a health care professional.^3,4,5^ A review of the Disneyland outbreak implicated personal belief exemptions as the reasons for undervaccination or nonvaccination in two-thirds of measles cases.^6^ In response, state lawmakers passed Senate Bill No. 277 (SB 277),^7^ which eliminated nonmedical exemptions to childhood immunizations before the 2016-2017 school year. The passing of SB 277 made California the first state in more than 30 years to eliminate nonmedical exemptions,^3^ thereby providing an opportunity to better understand both the intended and unintended consequences of such a policy change. School entry mandates and vaccine exemptions have been an active area of state policy and legislation,^8^ and many states have looked to California as an example when drafting similar legislation.

After the implementation of SB 277, data from the California Department of Public Health^9,10^ showed that the law proved effective in increasing vaccine uptake, with kindergarten immunization rates increasing from 92.8% in the 2015-2016 school year to 95.1% in the 2017-2018 school year.^11^ However, investigators were concerned that the law created an incentive for parents who could no longer obtain a nonmedical exemption to either substitute a medical exemption^12^ or to homeschool their children and thereby circumvent school entry immunization requirements (a new provision of SB 277).^13^ In fact, the medical exemption rate for kindergarteners almost tripled from the 2015-2016 to 2017-2018 school years^11,14^; when many of these exemptions were suspected of being fraudulent, the legislature passed Senate Bill No. 276 in 2019 to strengthen the requirements to obtain a medical exemption.^15^ Although removal of unvaccinated children from congregate settings such as school and daycare may reduce the risk of highly transmissible vaccine-preventable diseases, it may also have consequences on children’s social and emotional development, given differing opportunities for peer interaction.^16^ The purpose of this study was to evaluate the second potential mechanism to bypass vaccination by describing the changes in homeschooling rates before and after SB 277.

## Methods

Parents choosing to homeschool in California may do so by one of several mechanisms, including independent study programs (ISPs), private school satellite programs (PSPs), or a private school affidavit (PSA) (Table 1).^17^ We herein describe our approach to estimating enrollment for each homeschooling mechanism from the 2012-2013 to 2019-2020 school years. Data were collected for this study from October 3, 2012, to October 2, 2019. The study did not require institutional review board approval because we used only public databases from the California Department of Education (CDE) with deidentified information; furthermore, we had no interaction or intervention with the population being studied. This study followed the Strengthening the Reporting of Observational Studies in Epidemiology (STROBE) reporting guideline.^18^

**Table 1.** Overview of the Major Homeschooling Mechanisms in California

Homeschooling mechanism	Type of school	Curriculum support	Administrative support	Fee	Secular	Reasons parents may choose option
Traditional ISP	Public	Yes	No	No	Yes	To provide a structured education mirroring classroom-based instruction with a centralized curriculum comprising the recommended core subjects
Charter school–based ISP	Public	Yes (parents may choose to receive a stipend and purchase their own curriculum)	No	No	Yes	To provide a structured education usually adhering to a particular educational philosophy (eg, Montessori and/or Waldorf)
PSA	Private	No	No	No	No	To provide an individualized education at the parents’ discretion
PSP	Private	Variable	Yes	Yes	No	To provide a coordinated education that caters to a parent’s and/or a child’s specific needs (frequently parochial)

Abbreviations: ISP, independent study program; PSA, private school affidavit; PSP, private school satellite program.

### Independent Study Program

An ISP is a program of the public school system in California. Compared with other homeschooling mechanisms, an ISP offers the greatest support to parents, by providing either a standardized curriculum and educational material or by providing funding so that parents may purchase their own curriculum.^19,20^ Parents may choose an ISP because their child has competing priorities (eg, a child actor, professional athlete, or child with health issues) or requires an adjusted curriculum (eg, the child requires specialized attention or qualifies for accelerated coursework), among other reasons.^19^ Students enrolled in an ISP are required to meet with a credentialed teacher regularly and take school- and state-administered tests.^20^ To identify ISP programs in California, we obtained a list of all past and current public schools from the CDE.^21^ The CDE obtains enrollment data for all schools for kindergarten through grade 8 (K-8) on Census Day, or the first Wednesday in October. Independent school programs were identified as schools that offered any combination of grades inclusive of K-8 in at least 1 school year from 2012-2013 to 2019-2020 and that were either primarily virtual (defined by the CDE as a school that provides virtual instruction but that may include some physical meetings between students and teachers) or exclusively virtual (defined by the CDE as a school where all instruction is virtual).^22^

### Private School Affidavit

Another mechanism for homeschooling in California is by filing a PSA^17,20,23^; this allows the family’s residence to become a standalone school with the parent acting as administrator and teacher. In this capacity, parents keep detailed records of attendance, coursework, and grades and provide instruction in a variety of core subjects.^23^ Private school affidavits receive no funding, curricular materials, or instructional support^24^ but afford a substantial degree of autonomy.^23^ Although the CDE publishes data on private school enrollment, these data are censored for schools with fewer than 6 students. To obtain the censored data, we filed a California Public Records Act request with the CDE for a deidentified listing of all schools with 5 or fewer enrolled students. We created 2 estimates of homeschooling enrollment via PSA: the low estimate, which included schools that had an enrollment of only 1 student, and the high estimate, which included schools that had enrollment of 5 or fewer students (a high estimate was created because we cannot exclude the possibility that some of the larger PSAs may represent very small brick-and-mortar private schools).

### Private School Satellite Program

A PSP is a private school that has filed an affidavit and whose main function is to support home-based instruction.^25,26^ Private school satellite programs may require parents to serve as teachers and/or administrators and provide administrative, curricular, and community support (including opportunities for students to socialize and engage in enrichment activities) in exchange for a membership fee.^17,25,27^ Some PSPs are denominational.^26^ California maintains a database of all private schools in the state^28^; however, the database does not distinguish between a brick-and-mortar private school and a PSP. To identify PSPs, we compiled a list from PSP directories maintained on the websites of 3 homeschooling networks (California Homeschool Network,^25^ A2Z Homeschooling,^24^ and Homeschooling Concierge^26^). To confirm that the listed schools were PSPs, we individually reviewed each school’s website and/or contacted the school administrators via telephone or email. Of note, 15 PSPs did not exist in the CDE database. An additional 9 PSPs were brick-and-mortar schools with a PSP offshoot; these were excluded because the proportion of the total enrollment that was accounted for by the PSP is unclear. To address uncertainty in PSP classification, we created 2 estimates of PSP enrollment: the low estimate, which included only schools that were confirmed as PSPs via website, telephone call, or email communication with a school official, and the high estimate, which also included schools that could not be confirmed as PSPs.

### Total Enrollment

The total number of students enrolled in grades K-8 in California was calculated by combining the count data from 3 separate databases: the public school database (available on the CDE public school website^21^), the private school database with 5 or fewer students (available from the CDE through a California Public Records Act request), and the private school database with greater than 5 students (available on the CDE private school website^28^). The CDE databases also included the category ungraded elementary, which represented students who did not fit into a particular grade level; these were included in the numerator for ISP, PSA, and PSP counts and in the denominator for total enrollment.

### Statistical Analysis

Data were analyzed from October 3, 2012, to October 2, 2019. We calculated homeschooling rates as the number of children enrolled through each of California’s 3 homeschooling mechanisms divided by all students enrolled in school in the same academic year. We calculated rates for each school year from 2012-2013 through 2019-2020 for the entire K-8 population, as well as grade-specific and homeschooling mechanism–specific (ISP, PSA, and PSP) rates. Rates were calculated for both low and high estimates of PSA and PSP enrollment as described earlier.

To assess the trends in homeschooling rates after the elimination of nonmedical exemptions to childhood immunizations for school entry, we conducted interrupted time series analyses using linear regressions, in which the outcome variable was the percentage of children enrolled in a homeschool program. Periods were defined as pre–SB 277 (academic school years 2012-2013 to 2015-2016) and post–SB 277 (academic years 2016-2017 to 2019-2020; dummy coded 0 and 1, with 0 being pre–SB 277), given the 2016 implementation date of the law. Other variables included in the model were academic year as well as a composite variable of homeschooling mechanism (ISP, PSA, and PSP) and grade level (K-8), which was used as a random intercept to account for previous trends in homeschool rates. To ensure that variance was not underestimated, robust SEs were used in the analysis. The significance level was set at .05 and the hypothesis tests were 2 sided. Of note, stronger methods (eg, synthetic control or controlled interrupted time series) were considered and would be relevant if other states’ homeschooling rates were declining; however, homeschooling rates are increasing nationally^29^ and so it was believed they would be unlikely to provide a different answer.

The outcomes for the interrupted time series analyses were as follows: low and high estimates for all children enrolled in all homeschool programs, low and high estimates for children enrolled in all homeschool programs by grade (each grade was analyzed separately), all K-8 children enrolled in ISPs, and children enrolled in ISPs by grade. Restriction of the sample to K-8 enrollment in ISPs alone evaluated the hypothesis that parents seeking to homeschool their children merely to bypass vaccination would be more likely to choose the option with the greatest degree of curricular or financial support. Restriction to kindergarten enrollment evaluated only the hypothesis that any effects of the elimination of nonmedical exemptions would be seen at the main entry point into schooling. Students already enrolled with a nonmedical exemption before SB277 were grandfathered in subsequent grades.^30^ Inferential analyses were conducted in Stata, version 16.1 (StataCorp LLC).

## Results

### Total Homeschooling Enrollment

**Figure 1.**
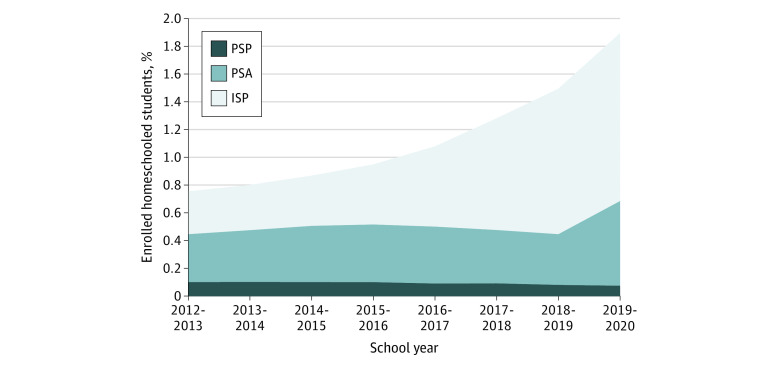
Trends in Homeschooling Rates in California for School Years 2012-2013 to 2019-2020 ISP indicates independent study program; PSA, private school affidavit; and PSP, private school satellite program.

Total homeschooling enrollment for K-8 students in California increased from 35 122 students or 0.8% of all enrolled K-8 students in the state in the 2012-2013 school year to 86 574 students or 1.9% during the 2019-2020 school year for the low estimate (42 379 students [0.9%] to 97 316 students [2.1%] for the high estimate). The results are presented in Figure 1.

### Homeschooling Enrollment by Grade Level

**Figure 2.**
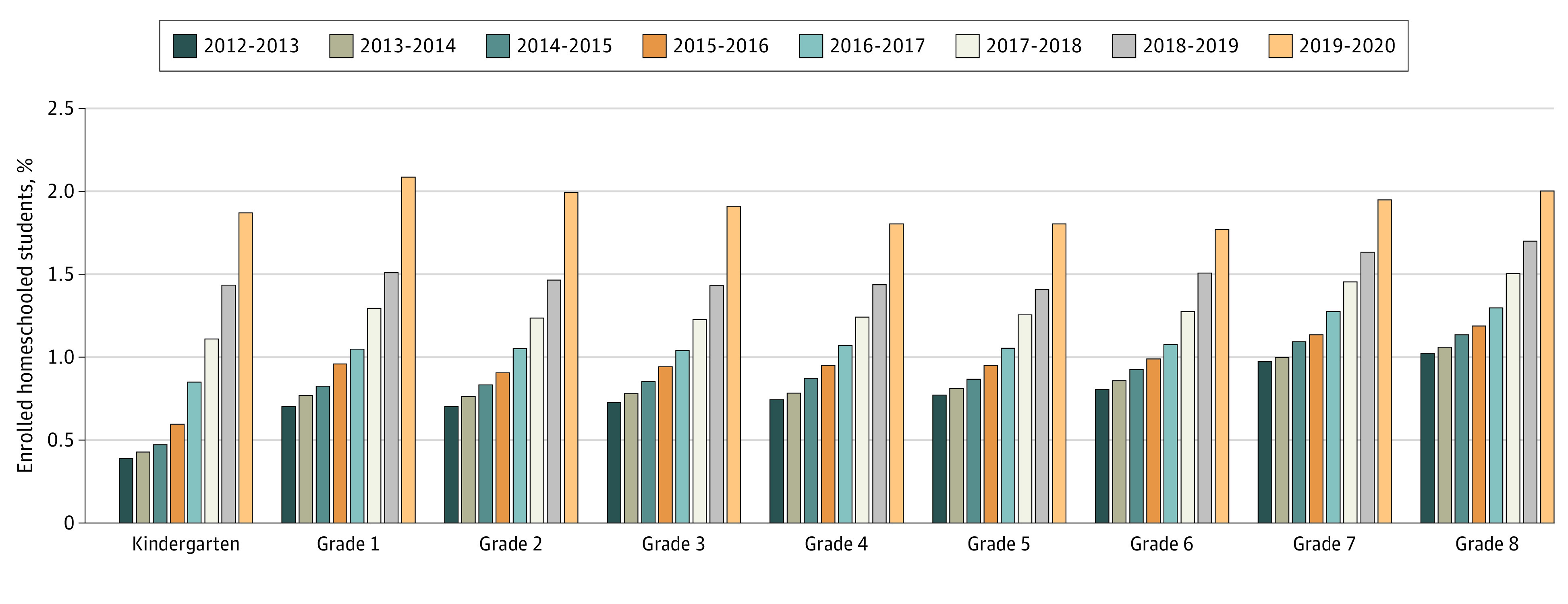
Total Percentage of Kindergarten Through Grade 8 Enrollment in Homeschool by School Year in California Data represent the low estimate.

The increase in homeschooling in California was greatest for the lower vs the upper grade levels (Figure 1); for example, kindergarten homeschooling enrollment increased from 2068 students or 0.4% of the total in the 2012-2013 school year to 10 553 students or 1.9% in the 2019-2020 school year (2231 students [0.4%] to 10 969 students [1.9%] for the high estimate), whereas grade 8 homeschool enrollment rate increased from 5146 students or 1.0% in the 2012-2013 school year to 10 485 students or 2.0% in the 2019-2020 school year (5263 students [1.0%] to 10 666 students [2.0%] for the high estimate). The results are presented in Figure 2.

### Homeschooling Enrollment by Mechanism

The mechanisms responsible for most homeschooling during the school year when SB 277 was enacted (2015-2016) included the following from most common to least common: for ISP, 20 149 students or 45.3% (20 149 students or 38% for high estimate); for PSA, 19 333 students or 43.5% (27 989 students or 53% for high estimate); and for PSP, 4733 students or 11.1% (4935 students or 9% for high estimate).

### Homeschooling Enrollment Before and After SB 277

We found no significant increase in the percentage of children enrolled in homeschooling programs in California associated with the implementation of SB 277 in 2016 (Table 2 and Table 3). However, when comparing pre–SB 277 implementation with post–SB 277 implementation, we did find small decreases in the percentage of change of students in all grade levels enrolled in ISPs (β = −0.007 [95% CI, −0.008 to −0.005]) (Table 2) and specifically for kindergarteners enrolled in ISPs (β = −0.008 [95% CI, −0.0010 to −0.006]) (Table 3). We also analyzed each of the other grade levels separately, and this small decrease was noted across all grade levels when assessing students enrolled in ISPs.

**Table 2.** Association Between the Elimination of Nonmedical Exemptions in California via SB 277 in 2016 and ISP, PSA, and PSP Enrollment for K-8 Students

Variable	β (95% CI)^a^		
	All homeschooling mechanisms		ISP only
	Low estimate	High estimate	
Constant	0.005 (−0.003 to 0.014)	0.010 (−0.011 to 0.032)	0.002 (0.001 to 0.003)
School year	0.001 (0.003 to 0.004)	0.001 (−0.007 to 0.009)	0.0003 (0.0003 to 0.0004)
Policy implemented	−0.001 (−0.024 to 0.022)	−0.001 (−0.054 to 0.052)	−0.007 (−0.008 to −0.005)
Year × policy	−0.0001 (−0.005 to 0.005)	0.001 (−0.012 to 0.011)	0.002 (0.001 to 0.002)

Abbreviations: ISP, independent study program; K-8, kindergarten through grade 8; PSA, private school affidavit; PSP, private school satellite program; SB 277, Senate Bill No. 277.

^a^ Results are coefficients for an indicator variable for the period after implementation of SB 277 in California from interrupted times series analyses (continuous outcome) of the homeschooling rate in California from school years 2012-2013 through 2019-2020.

**Table 3.** Association Between the Elimination of Nonmedical Exemptions in California via SB 277 in 2016 and ISP, PSA, and PSP Enrollment for Kindergarten Students

Variable	β (95% CI)^a^		
	All homeschooling mechanisms		ISP only
	Low estimate	High estimate	
Constant	0.001 (0.0001 to 0.001)	0.001 (0.001 to 0.002)	0.001 (0.001 to 0.002)
School year	0.0002 (−0.0001 to 0.0005)	0.0002 (−0.0001 to 0.0005)	0.0005 (0.0003 to 0.001)
Policy implemented	−0.004 (−0.008 to 0.0004)	−0.004 (−0.009 to 0.0002)	−0.008 (−0.010 to −0.006)
Year × policy	0.001 (−0.0003 to 0.0021)	0.001 (−0.0002 to 0.002)	0.002 (0.002 to 0.002)

Abbreviations: ISP, independent study program; PSA, private school affidavit; PSP, private school satellite program; SB 277, Senate Bill No. 277.

^a^ Results are coefficients for an indicator variable for the period after implementation of SB 277 in California from interrupted times series analyses (continuous outcome) of the homeschooling rate in California from school years 2012-2013 through 2019-2020.

## Discussion

To our knowledge, this study is the first to estimate homeschooling rates in California. We found no evidence that SB 277’s elimination of nonmedical exemptions to mandatory childhood immunizations for school entry before the 2016-2017 school year was associated with an increase in homeschooling rates for K-8 students in California. This finding suggests that SB 277 had minimal unintended consequences related to homeschooling.^3^ This may indicate that legislative action to limit exemptions to compulsory vaccination for school entry is not associated with removal from classroom-based instruction in brick-and-mortar institutions nor with restricted opportunities for peer interactions.

Our results are consistent with multiple explanations of parents’ responses to SB 277. One possibility is that vaccine-hesitant or vaccine-refusing parents who would have pursued a nonmedical exemption if available decided that the effort of homeschooling outweighed their opposition to vaccination. Alternatively, parents who would have pursued a nonmedical exemption, if available, may have instead pursued a medical exemption, an outcome consistent with the observed tripling of the medical exemption rate from the 2015-2016 school year (before SB 277) to the 2017-2018 school year (after SB 277), documented by our team.^14^ Another drastic mechanism for avoiding SB 277’s restrictions—moving out of the state—is difficult to document as a result of SB 277. Finally, parents may have avoided homeschooling as a solution to SB 277 simply because schools did not enforce SB 277 consistently. In previous work, we found evidence to suggest that the law was variably interpreted, implemented, and enforced across school districts, given vague regulatory language.^11,31^ One study found that the number of students overdue for vaccination more than quadrupled,^3^ suggesting that some parents were being allowed to enroll their children in brick-and-mortar schools despite being undervaccinated or unvaccinated.

It is important to note that other legislative, regulatory, and surveillance responses to increasing nonmedical exemptions in California may have created pressure to homeschool before SB 277. For example, Assembly Bill 2109 (passed in 2012 and implemented in 2014) tightened the criteria for obtaining a nonmedical exemption.^3^ In 2015, state and local health departments in California additionally began an effort to ensure proper application of the state’s conditional school entrance criteria for students not up to date with vaccinations.^3^ If this was the case, increases in homeschooling rates may have occurred for several years rather than immediately after the implementation of SB 277, creating the possibility of a type II error in our analysis. However, we find this explanation unlikely given that before SB 277 was implemented, children who were homeschooled via either public or private homeschooling options were subject to the same school entry immunization mandates and had the same nonmedical exemptions options available to them.

### Limitations

This study has an important limitation: Although we are confident that the ISP and PSA data are complete and accurate, PSP data are subject to reporting biases inherent in crowdsourced listings on websites. Additional possible sources of error include PSPs that were either not registered with the CDE (and had no publicly available data) or PSPs that were affiliated with brick-and-mortar private schools (and had merged data). Given the relatively small contribution of PSPs to overall homeschooling rates (approximately 10%), we do not believe the uncertainties regarding PSP enrollment had a significant impact on the analysis.

## Conclusions

The findings of our evaluation of homeschooling trends in California before and after SB 277 suggest that the elimination of nonmedical exemptions to mandatory childhood immunizations for school entry is not associated with an increase in homeschooling rates. This conclusion is predicated on the continued ability of vaccine-refusing parents to enroll children in brick-and-mortar schools, either by substituting with a medical exemption or by residing in a local school district with poor implementation of the law. States looking to eliminate nonmedical exemptions to childhood immunizations can learn from California’s example to better understand the unintended consequences of elimination of nonmedical exemptions to childhood immunizations; they may then use this insight to craft legislation capable of realizing the largest gains in vaccination rates with minimal unintended consequences.
